# The Lattice Kinetic Monte Carlo Simulation of Atomic Diffusion and Structural Transition for Gold

**DOI:** 10.1038/srep33128

**Published:** 2016-09-15

**Authors:** Xiang He, Feng Cheng, Zhao-Xu Chen

**Affiliations:** 1 State Key Laboratory of Lake Science and Environment, Nanjing Institute of Geography and Limnology, Chinese Academy of Sciences, Nanjing 210008, China; 2 Key Laboratory of Mesoscopic Chemistry of MOE, Institute of Theoretical and Computational Chemistry, School of Chemistry and Chemical Engineering, Nanjing University, Nanjing 210093, China

## Abstract

For the kinetic simulation of metal nanoparticles, we developed a self-consistent coordination-averaged energies for Au atoms based on energy properties of gold bulk phases. The energy barrier of the atom pairing change is proposed and holds for the microscopic reversibility principle. By applying the lattice kinetic Monte Carlo simulation on gold films, we found that the atomic diffusion of Au on the Au(111) surface undergoes a late transition state with an energy barrier of about 0.2 eV and a prefactor between 40~50 Å^2^/ps. This study also investigates the structural transition from spherical to faceted gold nanoparticles upon heating. The temperatures of structural transition are in agreement with the experimental melting temperatures of gold nanoparticles with diameters ranging from 2 nm to 8 nm.

Recently much attention has been paid to metal nanoparticles, owing to their unique thermodynamic, electrical, magnetic, optical, chemical and catalytic properties, which strongly depend on the particle size and geometries^1^,^2^. Gold nanoparticles have been extensively studied with a focus on their newly uncovered catalytic properties^3^. For example, gold nanoparticles play an important role in chemical engineering and materials science for their superior features, which can be used in the fields of industrial catalysts, clean energy and environment^4^,^5^,^6^,^7^. Researchers have found that the morphology and the size of particles are crucial to the physical and chemical properties of nanoparticles^6^,^8^,^9^. Because of the sensitive relationship between the properties, size and morphology of the nanoparticle, and the tendency for particles to aggregate due to their high surface energies, various methods have been developed to control gold nanoparticle morphology and size^2^. Naturally, the investigations of the dynamics of the nanoparticles are very important for the proper preparation and stabilization of nano-materials with specific properties by tuning their shape and size. However, it is still a challenge to directly observe the formation and transformation of the metal nanoparticles even with advanced physical microscopic imaging techniques, such as STM and AFM^7^,^8^,^10^,^11^. Therefore, electronic structure calculations and atomistic simulations have been widely used in the study of gold nanoparticles in addition to the experimental observations at a nanoscopic scale.

First-principles density functional theory has been successfully applied to the metal system, but the typical studies are on the small clusters due to the limitation of computation resources^12^,^13^. The kinetic Monte Carlo (kMC) method can simulate a larger temporal step than the fine temporal step produced by the molecular dynamics method, which makes it suitable to study the long-time evolution of large nano-metal systems containing numerous atoms^14^,^15^,^16^. The reliability and accuracy of the kMC simulation are determined by the calculation of all transition rates, which can be computed from the interatomic interaction and the energy barriers of transitions.

Chen and Wang developed a simple interatomic interaction model for solid solution where the pair interaction between nearest lattice sites was considered^17^. The utilized pair interaction was derived from physical parameters of the metals without semi-empirical parameters and was successfully applied to study the micro alloys^17^,^18^. However, the utilized pair interactions considered only the type of pairing atoms and ignored of the coordination number. This simplification leads to problems when the study turns from a uniform solid solution to the nanoparticles, where the studied atoms on surfaces are much different from the inner ones. Therefore, a developed pair interaction model that can handle atoms in different coordination environment such as at corners and edges is necessitated by the simulation of faceted metal nanoparticles. In addition, a reasonable analytical approach to calculate the energy barrier is extremely crucial to the kinetic simulation of nanoparticles.

Recently we proposed an equation to calculate the energy barriers based on atomic interaction^19^. In this work, an interatomic interaction model distinguishing the atomic coordination number is developed and the calculation method of energy barriers depending on the location of the transition state is introduced. The coordination-averaged energies of Au atoms were derived and applied to study the diffusion of Au adatom on Au(111) and structural transition of gold nanoparticles.

## Model and Theoretical Methods

### Model and kMC algorithm

The initial Au nanoparticle is modeled in a rigid three-dimensional hexagonal cell of lattice sites. Each lattice site is either unoccupied or occupied by one Au atom. The nearest distance between two Au atoms is 2.8 Å. One Au atom only interacts with the other atoms at the nearest distance and belongs to the same cluster. The gyration radius of Au nanoparticle, *R*_*g*_, is defined as


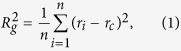


where *n* is the number of atoms in the cluster, *r*_*i*_ is the coordinates of the *i*th atom in the cluster and *r*_*c*_ is the center of mass of the cluster. The diameter of nanoparticle *D* is defined as 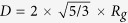.

In each kinetic Monte Carlo step, one gold atom of the cluster either tries to move spontaneously to an empty neighbor site or is pushed to the neighbor empty sites by other neighbor gold atoms.

The attempting movement has the time of occurrence Δ*t* = −log *u*/*r*, where *u* is a random number between 0 and 1 and *r* is the rate constant for the movement. The rate constant *r* at temperature *T* is determined by the Arrhenius equation


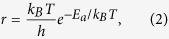


where *E*_*a*_ is the the energy barrier of the attempting movement of the atom and *k*_*B*_ is the Boltzmann constant.

In this study, we adopted the algorithm of the first reaction method which accepts the movement with the smallest time of occurrence Δ*t* among all possible movements^20^,^21^.

### Atomic coordination-averaged energy

With the lattice model, each atom of the nanoparticles is paired with coordinated atoms. We define the atomic coordination-averaged energy of Au atom, *I*^*z*^, in the *z*-fold coordinated Au bulk as


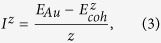


where *E*_*Au*_ is the energy of an isolated Au atom and 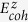 is the cohesive energy of Au bulk. The cohesive energy is defined by


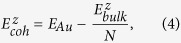


where 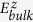 is the total energy of the bulk and *N* is the number of atoms in the bulk. Since each atom in the bulk is *z*-fold coordinated,


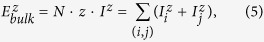


where (*i*, *j*) are the pairs of neighboring atoms in the bulk. Clearly, the bulk energy is also the sum of the interatomic interactions over all pairs in the bulk and the pair interaction is the sum of coordination-averaged energies of the two pairing atoms.

We extend Eq. (5) to any independent metal cluster and assign the pair interaction to the two pairing atoms according to the coordination number:


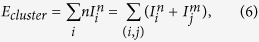


where the atom *i (n*-fold coordinated) and the atom *j (m*-fold coordinated) are paired in the cluster. Thus, the total energy of the cluster can be calculated by the coordination-averaged energies sum of all atoms.

It should be mentioned that when the pair (*i*, *j*) is broken, the energy change is not 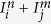. The energy to break the pair is


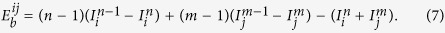


The first two items in the rhs. of Eq. (7) are the energy change due to the decrease of the coordination number of the atoms. If only one atom, such as *i*, is coordinated after the pair is broken, the Eq. (7) can be cast into


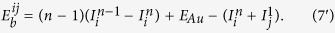


If various pairs are broken simultaneously, Eqs (7) and (7′) can be applied continuously once for one broken pair to calculate the total energy change. Similarly, the energy to form one or various pairs can be calculated by applying Eq. (8).


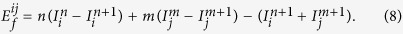


According to Eqs (7) and (8), both 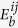 and 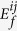 are positive. We define 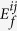 to be positive only for the sake of convenience, though conventionally it is negative. Once knowing the coordination-averaged energies of Au atoms with different coordination numbers, one could calculate the energy of Au nanoparticles for kMC simulation. The determination of *I*^*z*^s is presented in the discussion section. Before that, the calculation of the energy barrier, which is crucial to the kMC simulation, is discussed in the following section.

### Energy barrier of pairing change

In our simulation, the movement of one Au atom in the nanoparticle is attempted for each kMC step. The movement involves the breaking and formation of various Au-Au pairs. For large nanoparticles, it is hard to compute the energy barrier of the movement using the first-principles density functional theory. An reliable approach holding for the microscopic reversibility principle is highly desired to calculate the barriers *E*_*a*_.

Generally, the energy barrier *E*_*a*_ should be between Δ*E* and *E*_*b*_ for endothermic reactions or between 0 and *E*_*b*_ for exothermic reactions, where Δ*E* is the total energy change and *E*_*b*_ is the total energy to break Au-Au pairs in the movement, respectively. The total energy change Δ*E* = *E*_*b*_ − *E*_*f*_, where *E*_*f*_ is the absolute value of the total energy to form Au-Au pairs. Thus a possible expression satisfying the microscopic reversibility principle is


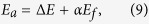


where the co-efficient *α* (0 ≤ *α* ≤ 1) characterizes the location of the transition state along the reaction coordinate. As a coarse approach, we take unitary *α* in Eq. (9) and obtain 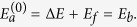 Clearly 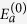 represents the energy needed to break Au-Au pairs in the attempting movement.

The energy barrier in our previous study^19^ was calculated as


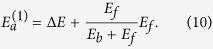


Compared with 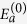, the fractional *α* makes the transition state move towards the final state. The energy barrier with even small *α* that fits the principle of microscopic reversibility are also made available by


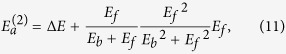



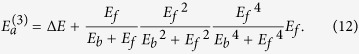


Obviously, these energy barriers with small *α* correspond to the late transition states.

The choice of Eqs (10) to (12), 22, 23 depends only on the position of the transition state along the reaction coordinate. Once *E*_*b*_ and *E*_*f*_ are known, the calculation of the energy barriers does not require any empirical parameter, which is usually present when applying the Brönsted-Evans-Polanyi principles^22^,^23^. Furthermore, the movement of Au atoms in different coordination environments, such as the corner, edge and surface of the nanoparticle, involves different Au-Au pairs; thus, the calculated *E*_*a*_ and rate constants of the Au movements are distinguished as a consequence in our model.

## Results and Discussion

### The coordination-averaged energies of Au atoms

The bulk gold is the hexagonal close packing structure. The Au atom in the bulk has the coordination number of 12 and a cohesive energy of −3.81 eV^24^. To calculate *I*^*z*^(*z* < 12) by Eq. (3), one should know 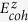 for other gold bulk phases. Järvi *et al.* calculated the cohesive energies of Au bulk for diamond, simple cubic and body centered cubic phases based on the ReaxFF framework^25^. Meanwhile, the cohesive energy of one-fold coordinated Au can be derived from the dissociation energy of an Au dimer^26^. Thus, the cohesive energies of 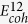, 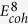, 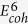, 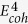 and 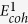 are obtained from the literatures. But a lack of cohesive energies remains a problem for other coordination numbers.

Guevara *et al.* found that the cohesive energy depends on coordination with the power of 2/3^27^. Ibach pointed out that the binding energy of atoms can be fitted with a fractional exponent of the coordination number of 0.3 according to the effective medium theory at the lowest level of approximation^28^. Taking these findings into account and by the definition of Eq. (3), we proposed that the coordination-averaged energies of Au can be expressed as a function of coordination number *z* by


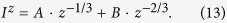


Now all *I*^*z*^ can be obtained by fitting known 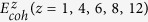 using Eq. (13). It worth noting that *E*_*Au*_ is unknown in our model and needs to be worked out.

Since the sum of the cohesive energy and the formation energy of vacancy 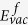, which is 0.89 eV for the Au bulk^29^, is equal to the energy change of the removal of one atom from the bulk, the following equation holds in our model from Eqs (7) and (8):


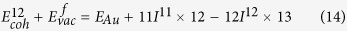


Such that *I*^*z*^ and *E*_*Au*_ are iterative solutions to Eqs (3), (13) and (14). By initializing *E*_*Au*_ to some value, *I*^*z*^(*z* = 1, 4, 6, 8, 12) are determined by Eq. (3) and *I*^11^ is fitted by Eq. (13). From Eq. (14), *E*_*Au*_ is calculated and used to determine *I*^*z*^ again until *E*_*Au*_ is converged. The converged solutions of *E*_*Au*_ and the coordination-averaged energies of 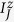 are listed in Table 1.

**Figure 1:**
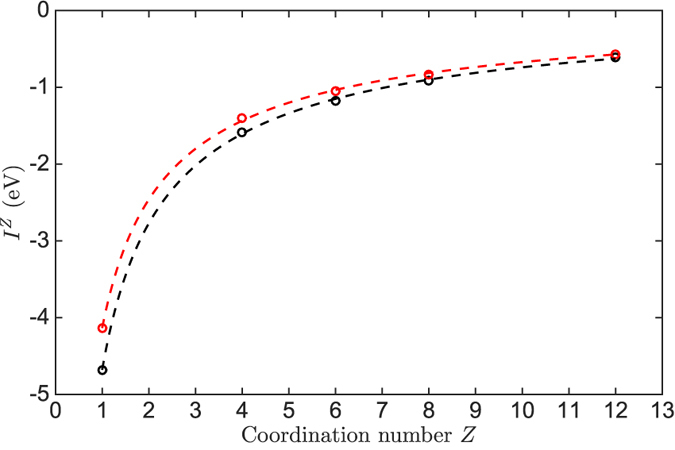
The dash lines are the fitting curves of the coordination energies by Eq. (13). The black dash line is with *E*_*Au*_ of −3.53 eV and the red one is with *E*_*Au*_ of −2.99 eV. *I*^*z*^(*z* = 1, 4, 6, 8, 12) calculated by Eq. (3) with two different *E*_*Au*_s are presented by the black and red open circles respectively.

Recently, Backman *et al.* developed a bond order potential for gold and also calculated the cohesive energies of the diamond, simple cubic and body centered cubic Au bulk phases^30^. The atomic coordination-averaged energies derived from their results, 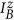, is also listed in Table 1. The two series of *I*^*z*^ as a function of coordination number are drawn in Fig. 1. It is clear that the calculated *I*^*z*^(*z* = 1, 4, 6, 8, 12) with solved *E*_*Au*_ by the definition Eq. (3) are very close to the fitting curves, which means that the calculation results, without extra empirical or semi-empirical parameters, are self-consistent in our model.

The two series of *E*_*Au*_ and *I*^*z*^ of low-coordinated Au in Table 1 are somewhat different from each other. The difference of *E*_*Au*_ is 0.54 eV. To compare the influence on results with the two series of *I*^*z*^, we calculated the desorption energy *E*_*d*_ of the single Au adatom, which desorbs from the Au(111) surface and involves *E*_*Au*_ as well as the low-coordinated *I*^3^. From Eqs (7) and (7′), we have


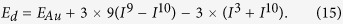


Using Eq. (15), the estimated *E*_*d*_ are 2.86 and 2.53 eV respectively. We performed further DFT calculation and found the desorption energy of the single Au atom was 2.54 eV, which is between the two estimated *E*_*d*_. Interestingly, although the estimation of the desorption energy by our method is much simpler than the extensive DFT calculation, the results are numerically comparable. Since the estimated *E*_*d*_ using 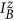 is much close to the DFT result, 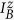 is used throughout the following studies.

### Atomic diffusion of Au on Au(111) surface

Compared with the Monte Carlo method, the kinetic Monte Carlo method can simulate the evolution of processes, which makes the study on the physic properties related to time possible. In this section, the dynamic simulations of the Au atom on the Au(111) surface were performed from 200 K to 700 K using the energy barriers in Eqs (10)~(12) and two series of coordination-averaged energies in Table 1. The rational position of the transition state along the reaction coordinate of the gold atom movement is examined by the behavior of atomic diffusion of Au.

**Figure 2:**
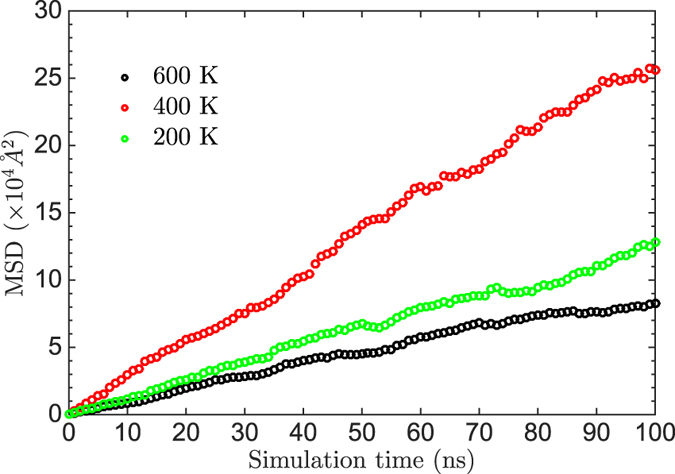
The mean-square displacement of Au vs time at different temperatures with 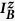 and using the barrier 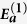 at 600 K, 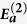 at 400 K and 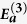 at 200 K.

The mean-square displacement (MSD) is defined as |*R*(*t*) − *R*(0)|^2^, where *R*(0) is the initial position of the diffusing Au atom and *R*(*t*) is the position at time *t*. Typical behaviors of MSD in our simulation results are illustrated in Fig. 2, which is simulated using 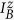 in Table 1.

In our study, one diffusion simulation is repeated 100 times at a constant temperature. Thus, each point in Fig. 2 is the average of 100 simulations. The MSD increases linearly with the time, and the diffusion coefficient *D* can be extracted from the slope of MSD to time according to Einstein relation:


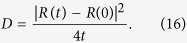


**Figure 3:**
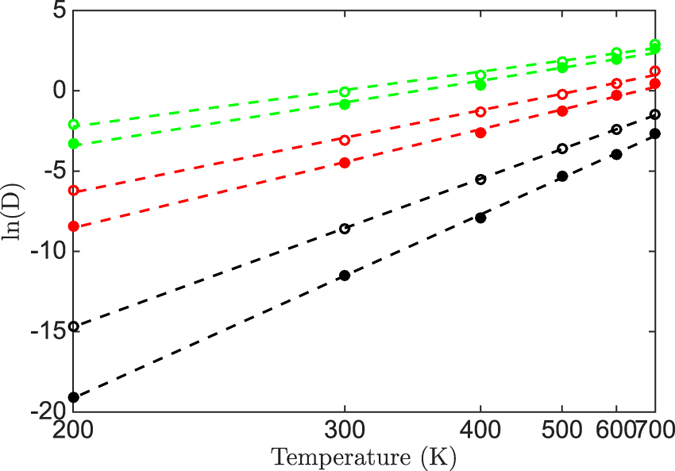
The logarithmic diffusion coefficient vs reciprocal temperature. The solid circles and open circles represent the diffusion coefficient simulated using the 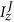 and 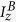 respectively. The circles in black, red and green are simulated with the barriers of Eqs (10)~(12), respectively, and fitted by dash lines.

The relations between the diffusion coefficient, simulated with different barriers, and the temperature are presented in Fig. 3. As can be seen, *D* follows the well known Arrhenius relationship


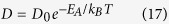


in the temperature range of simulations, where *D*_0_ is the pre-exponential factor and *E*_*A*_ is the activation energy of the diffusion.

The calculated *D*_0_ and *E*_*A*_ with different barriers and coordination-averaged energies are listed in Table 2.

The experimental measurement of the diffusion of Au adatoms on the Au(111) surface is quite rare. Jaklevic and Elie have reported the scanning tunneling microscope (STM) observation of surface diffusion of Au on a clean and annealed Au(111) surface^31^. Lin and Chung have measured the gold surface diffusion activation energy of ~0.22 eV using STM in a narrow range of temperatures^32^. From Table 2, one can find that the simulation with 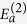 produces reasonable activation energies (0.212 and 0.177 eV) coinciding with the experimental measurement. Clearly, the atomic diffusion simulated by 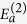 is easier (harder) than by 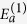 (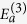), which indicates a late transition state along the reaction coordinate. To our knowledge, no further experiment results have been reported and only theoretical results of the pre-exponential factor are available due to the surface reconstruction of Au(111)^33^.

Generally, theoretical studies estimate that the barrier to hopping of the Au adatom on Au(111) surfaces is not larger than 0.2 eV and even smaller in some literatures^33^. Using the embedded atom method (EAM), Boisvert and Lewis studied the homodiffusion of single adatoms on a flat Au(111) surface and found *D*_0_ of 0.6 Å^2^/ps and *E*_*a*_ of 0.014 eV^34^. However, their first-principles calculation found that the energy barrier for adatom homodiffusion on Au(111) surface is 0.22 ± 0.03 eV, which is much larger than the results of EAM^35^. Within a large range of temperatures, Fernando and Treglia reported *E*_*a*_ of 0.12 eV and *D*_0_ of 3.4 Å^2^/ps by the molecular dynamic simulations of diffusion of Au on Au(111) with many-body tight-binding potentials^36^. Agrawal *et al.* investigated the hopping self diffusion on Au(111) surfaces using the Monte Carlo variational transition state theory and the Lennard-Jones interactions and found an *E*_*A*_ of 0.12 eV and *D*_0_ of 0.85 Å^2^/ps^37^.

The prefactors in our simulations are one order of magnitude larger than the existing calculation results^36^. It is worth noting that those large prefactors (which means the long-distance diffusion) are accompanied by small diffusion barriers (which means the easy diffusion). Since our simulation determines a larger diffusion barrier than those simulations, the corresponding perfactors should also be larger to produce the similar diffusion behavior^34^,^36^,^37^. In fact, Liu *et al.* have pointed out that prefactors of single adatoms of FCC metals are in the range of 1~100 Å^2^/ps by EAM study^38^. Furthermore, Ibach considered that the prefactor of surface diffusion on metals should be about 10 Å^2^/ps according to the transition state theory^28^. Thus, our results of *D*_0_ (42.3 and 49.9 Å^2^/ps) are reasonable.

Through our simulations, we found that the diffusion of an Au adatom on Au(111) undergoes a late transition state and the Eq. (11) can produce feasible diffusion barriers to simulate Au-Au pairs.

### Nanoparticle structural transition

The transformation of gold nanoparticles involves more types of formed and broken Au-Au pairs than the atomic diffusion on the surface. Thus we simulated the transformation of nanoparticles under heating to inspect the developed coordination-averaged energies and the energy barrier. The spherical FCC nanoparticles with diameters between 22 and 80 Å were built on the rigid lattice and heated from 200 to 1400 K. The temperature was increased by 10^−3^ K per Monte Carlo step. The simulation was preformed with the coordination-averaged energies 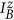 in Table 1 and the barrier 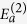. The root of mean-square displacement (RMSD) was used to observe the transformation of the heated gold nanoparticles.

**Figure 4:**
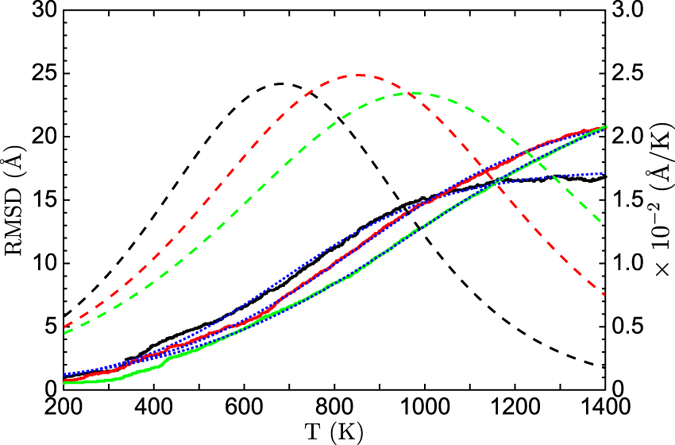
The RMSD of the heated 34 Å, 43 Å and 53 Å nanoparticles as a function of temperatures are shown in black, red and green lines, respectively, which are fitted in blue dotted lines. The corresponding derivatives of RMSD with respect to the temperature are shown in black, red and green dash lines.

Some instantaneous RMSDs with respect to the temperature during the heating of gold nanoparticles are shown in Fig. 4. It worth noting that the elapsed time of each Monte Carlo step at low temperatures is longer than at high temperatures which makes the heating at low temperatures far slower than at high temperatures. During the heating, the RMSD oscillated around small values under 300 K for long periods of time, then rose linearly with the increasing temperatures in short periods of time. At high temperatures, the RMSD should approach a constant value since the atoms cannot depart from the finite volume of the nanoparticles before the evaporation takes place. The RMSD as a function of temperatures can be well shaped by the sigmoid function as shown by the blue dotted lines in Fig. 4. The observed behavior of RMSD in Fig. 4 is similar to the characteristic of the transition from a solid phase to a liquid-like phase in MD simulations^39^. Lewis *et al.* have found that small gold clusters can undergo several structural transitions as a function of temperature^40^. However, it is hard to define the melting of nanoparticles in the rigid lattice model since the interaction and the distance of two neighbor atoms are fixed, which are flexible in off-lattice MD simulations. Nevertheless, we can locate the temperature of structural transition of the nanoparticle by finding the extreme point of the derivative of RMSD with respect to the temperature which are shown by the dash lines in Fig. 4.

**Figure 5:**
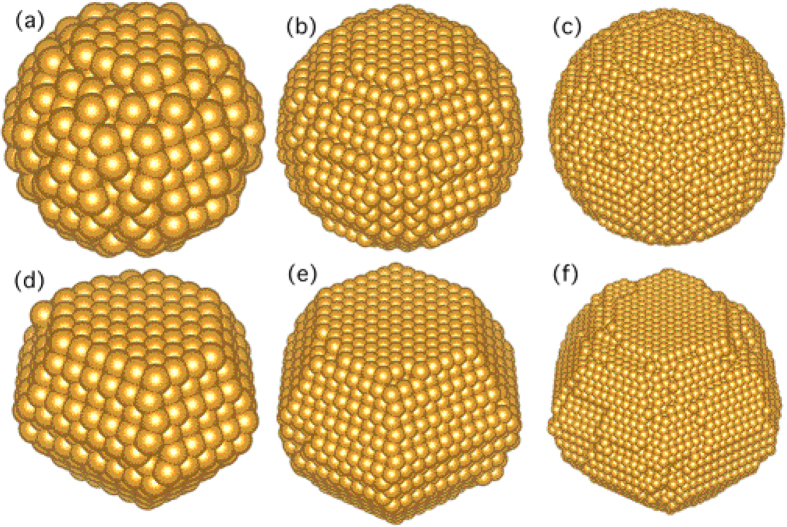
The initial spherical structures at 200 K of gold nanoparticles with diameters of (**a**) 34 Å, (**b**) 53 Å and (**c**) 80 Å. The respective structures at transition temperatures of (**d**) 691 K, (**e**) 988 K and (**f**) 1077 K after heating.

In this way, the transition temperatures *T* ^*^ of nanoparticles with diameters between 22 and 80 Å are determined in the range of a liquid-like phase. Compared with initial sphere structures, the gyration radius of simulated nanoparticles at *T* ^*^ increased by ~0.1 Å, which indicates that the morphology of nanoparticles becomes less spherical. The structures of the gold nanoparticles with diameters of 34 Å, 53 Å and 80 Å at the transition temperatures are presented in Fig. 5 together with their initial spherical structures at 200 K. Notably, the initial structure undergoes surface reconstruction upon heating and the nanoparticles turn into faceted structures at the transition temperatures. The structural transition is expected since the spherical structure is far from the equilibrium structure and the nanoparticle will change towards the faceted structures upon heating^41^,^42^. Thus, *T* ^*^ corresponds to the transition from the spherical structure to the faceted structure. It has been observed in the MD simulations using the modified embedded atom method that the initial FCC spherical gold nanoclusters transformed to the faceted structure, followed by a transition near the melting point^43^. Backman *et al.* also found that the small gold clusters transformed from spherical to faceted clusters at elevated temperatures at the onset of melting^30^. We, therefore, expect the structural transition from spherical to faceted structures found here to be strongly related to the melting of nanoparticles.

**Figure 6:**
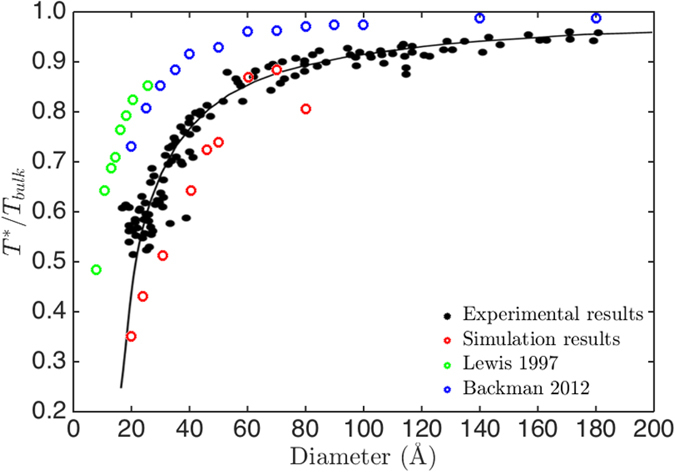
Relative structural transition temperature in the red open circles as a function of nanoparticle diameter. The relative melting temperatures by experiments are shown in black solid circles^44^. The temperatures by MD simulations are shown in the green and the blue open circles, respectively^30^,^40^. The plotted literature results are taken from ref. 30 with kind permission of The European Physical Journal (EPJ).

Both experimental and theoretical literature have found the well-known melting-point depression phenomenon of small nanoparticles, which melt at lower temperatures than bulk. The ratio of nanoparticle phase transition temperature, *T* ^*^, to gold bulk melting temperature is shown in Fig. 6. The experimental results and MD simulation results of melting temperatures are shown for comparison. Figure 6 exhibits a familiar behavior of melting-point depression of nanoclusters and approaches to unitary at large scale^30^,^40^,^42^,^44^. Backman *et al.* found that the simulated melting points of gold nanoclusters ranging from 2 to 18 nm are higher than experiments, which was attributed to the superheating^30^. Lewis *et al.* also reported notably higher melting points than experiments for gold nanoclusters smaller than 2.5 nm^40^. Compared with these results, the transition temperatures in our simulation are comparable to the experimental results of melting temperatures by Buffat and Borel^44^. The above results show that the adopted coordination-averaged energies and energy barrier are feasible and reasonable to simulate the structural transition of gold nanoparticles upon heating.

## Conclusions

The self-consistent coordination-averaged pairing energies of Au atoms are developed and applied in our kMC simulations on gold films and nanoparticles. Applying our proposed energy barriers based on the pairing change, we found that the atomic diffusion of Au on the Au(111) surface undergoes a late transition state along the reaction coordinate. A reasonable energy barrier of about 0.2 eV and a reliable prefactor between 40 and 50 Å^2^/ps are determined for the diffusion. Upon heating, the structural transition from spherical to faceted gold nanoparticles is investigated by kMC simulations as well. The simulated temperatures of structural transition are in agreement with the experimental results on the size dependence of gold melting temperatures. We expect that our proposed coordination-averaged energy model and the scheme to calculate the barriers are applicable and universal making it appropriate for studying other metal nanoparticles due to the simplicity of the method.

## Additional Information

**How to cite this article**: He, X. *et al.* The Lattice Kinetic Monte Carlo Simulation of Atomic Diffusion and Structural Transition for Gold. *Sci. Rep.* **6**, 33128; doi: 10.1038/srep33128 (2016).

## Figures and Tables

**Table 1:** The coordination-averaged energies of Au atoms with different coordination number (*z*).

*z*	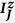 (eV)	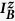 (eV)	*z*	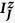 (eV)	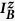 (eV)
*E*_*Au*_	−3.53	−2.99			
1	−4.68	−4.13	7	−1.01	−0.91
2	−2.71	−2.46	8	−0.90	−0.81
3	−2.02	−1.80	9	−0.81	−0.74
4	−1.61	−1.44	10	−0.74	−0.67
5	−1.34	−1.20	11	−0.68	−0.62
6	−1.15	−1.03	12	−0.63	−0.57

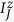
 and 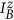 are derived with cohesive energies reported in refs 25 and 30 respectively.

**Table 2:** The pre-exponential factor (*D*_0_) and activation energy (*E*_*A*_) of Au on Au(111).

Barrier	With 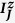		With 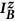	
	*D*_0_(Å^2^/ps)	*E*_*A*_(eV)	*D*_0_(Å^2^/ps)	*E_A_*(eV)
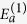	41.9	0.394	42.9	0.318
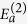	42.3	0.212	49.9	0.177
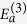	106.4	0.139	100.3	0.118
